# *In vivo* and *in vitro* evaluation of pharmacological activities of *Adenia trilobata* (Roxb.)

**DOI:** 10.1016/j.bbrep.2020.100772

**Published:** 2020-06-09

**Authors:** Niloy Barua, Md Arfin Ibn Aziz, Abu Montakim Tareq, Mohammed Aktar Sayeed, Najmul Alam, Nobi ul Alam, Mohammad Amran Uddin, Chadni Lyzu, Talha Bin Emran

**Affiliations:** a Department of Pharmacy, International Islamic University Chittagong, Kumira, Chittagong, 4318, Bangladesh; b Drug Discovery, GUSTO A Research Group, Chittagong, 4000, Bangladesh; c Biomedical and Toxicological Research Institute, Bangladesh Council of Scientific and Industrial Research (BCSIR), Dr. Qudrat-I-Khuda Road, Dhanmondi, Dhaka, 1205, Bangladesh; d Department of Pharmacy, BGC Trust University Bangladesh, Chittagong, 4381, Bangladesh

**Keywords:** *A. trilobata*, Analgesic activity, Antidiarrheal, Antioxidant, Cytotoxic, Thrombolytic, OS, oxidative stress, UV, ultra-violet, DPPH, 1,1-diphenyl, 2-picryl hydrazyl, A. trilobata, Adenia trilobata, LC_50_, 50% lethal concentration, IC_50_, 50% inhibitory concentration, FCR, Folin-Ciocalteu reagent, IP, intraperitoneal, SEM, standard error mean, ANOVA, Analysis of variance, b.w., body weight

## Abstract

*Adenia trilobata*, locally known as akandaphal in Bangladesh, has some traditional uses. Leaves and stems extracted with pure methanol (MEATL, MEATS) and fractioned by n-hexane (NFATL, NFATS), which was subjected to qualitative phytochemical analysis. The qualitative phytochemical analysis of four extracts showed the presence of secondary metabolites such as alkaloid, carbohydrate, glycosides, flavonoids, phenols, flavonol, and saponins. All four extracts of *A. trilobata,* exhibited a strong antioxidant activity while a moderately (MEATS = 328 μg/mL) to weakly toxic (NFATL = 616.85 μg/mL) LC_50_ observed in brine shrimp lethality bioassay. In thrombolytic test, MEATL (18.54 ± 2.18%; P < 0.01) and MEATS (25.58 ± 4.76%; P < 0.0001) showed significant percentage of clot lysis in human blood. The *in vivo* analgesic activity carried by acetic acid test and formalin test, while the antidiarrheal activity assayed by two standard methods e.g., castor oil-induced diarrhea and castor oil-induced gastrointestinal motility. Both, *in vivo* model, showed an extremely significant (P < 0.0001) dose-dependent manner percentage of inhibition in comparison to the control group. Present results suggested, *A. trilobata* could be a potential source for antioxidative, cytotoxic, thrombolytic, analgesic, antidiarrheal agents which require further study to identify the mechanism of *A. trilobata.*

## Highlights

• Detection of different plant metabolites in extracts of different plant parts.
• Strong antioxidant activity was found for all four extracts.
• Moderate to weakly toxic LC_50_ observed in brine shrimp lethality bioassay.
• A significant percentage of clot lysis in human blood was perceived.
• Extremely significant analgesic and antidiarrheal activity was found.

## Graphical abstract

**Figure:**
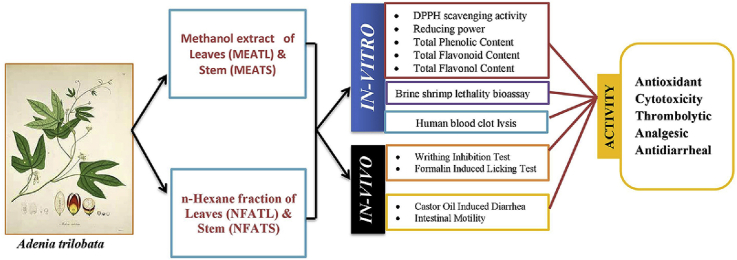

## 1 Introduction

The redox homeostasis plays an essential part in maintaining health and disease prevention. The imbalance of antioxidants and reactive oxygen species (ROS) is responsible for producing oxidative stress (OS) [1]. Free radicals initiate oxidative stress resulting in DNA damage and tissue damage which caused inflammation or cell death [2]. OS is associated with the prevalence of the cardiac disease, cancers, diabetes, neurodegenerative diseases, autoimmune disorders, aging, and others. The plant derives substances such as vegetables, and dietary fruits are rich in source of antioxidant. Antioxidant suggested having a significant benefit in health by reducing oxidative stress [1]. Thrombosis is a vital physiopathology which causes several atherothrombotic diseases (e.g., myocardial or cerebral infarction). The formation of a thrombus or blood clots in the artery because of the homeostatic imbalance leads to blockage of vascular organ and while recovering causes fatal significances, myocardial, or cerebral infarction, as well as death [3]. Pain is an unsavory phenomenon that comprises sensory experiences, including time, space, force, feeling, insight, and inspiration [4]. Several analgesic agents are isolated from natural sources such as morphine, aspirin [5]. Micro-organisms like *Salmonella*, *Escherichia coli*, *Vibrio cholera*, and Shigella are the most regular reasons for diarrhea in developing countries [6].

Biodiversity has a significant contribution to human livelihood. As per World Health Organization (WHO) reports, around 80% of the worldwide population still depends on herbal medications; today, several medicines owe their origin to medicinal plants [7]. Form the beginning of history; nature is the potential source for the drug substances. In the scientific community, the interest for new bioactive compounds from plant kingdoms is increasing day by day. In general, functional food or nutraceutical formulations could be useful in preventing several chronic diseases such as cancer, diabetes, a various inflammatory disorder, and obesity [8]. So, the international community encourages developing the naturally derived new compounds for the development of new drugs, which provide a tremendous pharmacological activity with lesser adverse effects and also less costly than available synthetic medicines [[9], [10], [11], [12]]. The cultivation of such plants, mainly if they are endemic, can be a potential income source for the developing countries. Thus, the disclosure of normal cures has additionally increased a great deal of consideration in these decades in the cosmetic sector. But, in some cases, modern science had not yet affirmed the ethnopharmacological used [13,14]. So, there is a strong need for the development of new cancer prevention agents, antinociceptive agent, and antidiarrheal agent from common natural sources for the development of novel drug products.

*A. trilobata* belongs to the Passifloraceae family, which is locally known as akandaphal. It is distributed in the Chittagong district of Bangladesh and also found in Andaman Is., Assam, East Himalaya, Myanmar, Pakistan, and West-Himalaya. *A. trilobata* has no exploratory work for human use; however, this plant utilized by the clans and nearby groups of the people for their medicinal services. A review found that the poultice of the leaves of this plant uses to treat headache, knee pain, snake bite, and stomach trouble [15,16]. In summary, there are no scientific reports on the biological activities of *A. trilobata*.

For these reasons, the present study figured to identify phytoconstituents and evaluate the antioxidant, cytotoxic, thrombolytic, analgesic, and antidiarrheal activities of methanol (MEATL, MEATS) and n-hexane (NFATL, NFATS) extract of *A. trilobata* leaves and stems*.*

## 2 Materials and methods

### 2.1 Chemicals

DPPH (1,1-diphenyl, 2-picryl hydrazyl), gallic acid, quercetin, sodium acetate, ferric chloride, and trichloroacetic acid obtained from Sigma Chemical Co. USA. Potassium ferricyanide, Folin-Ciocalteu reagent, aluminium chloride, sodium carbonate, and methanol purchased from Merck, Germany. Ascorbic acid purchased from SD Fine Chem. Ltd. India. Lyophilized streptokinase vial (1500000 IU), and vincristine sulfate (1 mg/vial) was purchased from Beacon Pharmaceuticals Ltd. Bangladesh.

### 2.2 Animals

Both sexes of Swiss albino mice weighing 25–35 gm. purchased from the Jahangirnagar University, Dhaka-1343, Bangladesh, at six-seven weeks old. The animals housed in standard conditions (room temperature 25 ± 2 °C; relative humidity 55–60%, 12 h light/dark cycle), with food pellets and water supply. The animals were adapted with the laboratory conditions for 14 days to use for the experiments. The study approved by the Institutional Animal Ethical Committee, Department of Pharmacy, International Islamic University Chittagong, Bangladesh according to governmental guidelines under the reference Pharm/PND/150/20–2019 [17].

### 2.3 Plant materials

Fresh leaves and stems of *A. trilobata* collected from the Hajarikhil Hill tract area, Chittagong, Bangladesh, in February 2019, which authenticated by Md. Anwarul Islam, Department of Botany, Jahangirnagar University, Savar, Dhaka-1342, Bangladesh under accession number Anwar-0311. After the collection of *A. trilobata*, it identified and confirmed by Professor Dr. Mohammed Aktar Sayeed, Department of Pharmacy, International Islamic University Chittagong, Kumira, Chittagong-4318, Bangladesh.

### 2.4 Preparation of methanol crude extract and n-hexane fraction

The leaves and stems dried under shade and ground for ten day's period, then dried in a mechanical drier at 60–70 °C. After drying, the leaves and stems were ground to a coarse powder and dissolved into methanol for 7 days. After that, the sediments filtered and dried in a water bath at 40–50 °C. A concentrated filtrate like the deep green color obtained after completely evaporating the solvent, which used as methanol extract (MEAT) for the experiment. Five (5 gm) of crude extract dissolved into water and methanol. The solution then was shaken well with n-hexane. The upper portion was then separated carefully by the separating funnel. The solvent was evaporated entirely with the help of the water bath and obtained n-hexane fraction (NFAT) used for the experiment.

### 2.5 Standardization and quality control of the extract

Methanol (MEATL, MEATS) and n-hexane (NFATL, NFATS) extract of *A. trilobata* leaves and stems was standardized and under-went quality control through physicochemical evaluation of crude extract, ensuring the safety and acute toxicity study in animal model [7].

### 2.6 Phytochemical screening

The phytochemical analysis of the methanol extract and n-hexane fraction of *A. trilobata* leaves and stems carried out the standard method to evaluate the alkaloid, carbohydrate, flavonoid, terpenoids, tannins, saponins, phenols, quinones, cholesterol, proteins, steroids, starch, sterols and flavonol [18,19].

### 2.7 Antioxidant activity

#### 2.7.1 DPPH free radical scavenging activity

Free radical scavenging activity of methanol extract and n-hexane fraction of *A. trilobata* leaves and stems, were determined by the method of Braca et al. [20]. The method based on the activity of scavenging the stable free radical 1, 1-diphenyl-2-picrylhydrazyl (DPPH). Three milliliter of 0.004% DPPH solution (4 mg DPPH in 100 mL of 95% methanol) added with the different concentrations (15.625 to 500 μg/mL) of the crude extract and the n-hexane fraction. The absorbance was taken at 517 nm after 30 min by the UV spectrophotometer.

(%) Radical scavenging = {(A_0_-A_1_)/A_0_} × 100

Where, A_0_ = absorbance of the control; A_1_ = absorbance of the extract.

Here, lower the absorbance values, the higher will be the free radical scavenging activity [21]. The IC_50_ (50% inhibitory concentration) was calculated as it indicated the effective concentration of the extract needed to scavenge 50% of the free radicals of DPPH.

#### 2.7.2 Reducing power capacity

Reducing power capacity was estimated by the method described by Oyaizu (1986) [22]. One milliliter of extract taken in serially diluted concentration (31.25, 62.5, 125, 250 and 500 μg/mL) and then added 2.5 mL phosphate buffer (0.2 M; pH 6.6) and potassium ferricyanide (1% w/v), respectively and incubated at 50 °C for 20 min to complete the reaction. After incubation, 2.5 mL trichloroacetic acid (10%) was added to the mixture and then centrifuged for 10 min at 3000 RPM, and 2.5 mL of the upper layer (supernatant solution) of the solution was withdrawn and added 2.5 mL distilled water and 0.5 mL FeCl_3_ (0.1% w/v), respectively. Then absorbance was taken at 700 nm by the UV spectrophotometer. If the absorbance of the reaction mixture increased with the increased of the concentration, then it was indicated the increased of the reducing power capacity. As a standard ascorbic acid and as a blank solution, phosphate buffer (0.2 M, pH 6.6) used.

#### 2.7.3 Total phenol content

The total phenol content of the extract was measured by using Folin-Ciocalteau reagent (FCR) as an oxidizing agent by the method of Singleton et al. [23]. 2.5 mL Folion-Ciocalteau reagent (FCR) (10 times diluted with water) and 2.5 mL sodium carbonate (Na_2_CO_3_) (20%) was mixed with 500 μg/mL extract. The mixture was made up to 10 mL by distilled water and incubated at 25 °C for 20 min to complete the reaction. The absorbance taken at 765 nm. The total phenol content concentration in the extract was then determined as mg of gallic acid equivalent by the equation obtained from the graph of standard gallic acid.

#### 2.7.4 Total flavonoid content

The total flavonoid content of the extract carried out by using a standard colorimetric method of Chang et al. using quercetin as standard [24,25]. In 500 μg/mL extract, 1.5 mL methanol and 100 μL aluminum chloride (AlCl_3_) (10%) was mixed. 100 μL potassium acetate (1 M) and 2.8 mL distilled water added into the mixture. The mixture incubated at room temperature for 30 min to complete the reaction. Then absorbance was taken at 415 nm against a blank solution containing all the reagents except extract. A standard quercetin graph determined the total flavonoid content and expressed as mg of quercetin equivalent concentration.

#### 2.7.5 Total flavonol content

The total flavonol content determined by adopting the method described by Kumaran and Karunakaran [26]. 500 μg/mL extract mixed with 0.5 mL AlCl_3_ (5%, 20 gm/L) and 1 mL sodium acetate (50 gm/L) solution. For completing the reaction, the mixture incubated for 150 min at room temperature, and then absorbance was taken at 440 nm against a blank solution containing all the reagents except the extract. Total flavonol content calculated as mg/g of quercetin equivalent by using the equation obtained from the standard quercetin graph.

### 2.8 Brine shrimp lethality bioassay

The brine shrimp lethality bioassay of *A. trilobata* observed by using simple organism *Artemia salina* leach (saline arrangement shrimp eggs). In the artificial seawater (3.8% NaCl solution), the shrimp eggs hatched for 48 h for maturing the shrimp called nauplii. The cytotoxicity bioassay carried on brine shrimp nauplii following the method described by Meyer et al. [27,28]. The extract was dissolved in DMSO (50 μL in 5 mL solution) to prepare the test sample with artificial seawater (3.8% NaCl in water) to obtain the serially diluted concentrations of 31.25, 62.5, 125, 250, 500 and 1000 μg/mL. Vincristine sulfate used as a positive control as the preceding method in a serial concentration dilution 0.125, 0.25, 0.5, 1, 5, and 10 μg/mL. Ten of the living nauplii applied to each of all experimental vials and control vials. Following 24 h, all vials inspected by an amplifying glass, and the number of living nauplii in each vial was observed and recorded.

% of mortality = (N_0_–N_1_/N_0_) × 100

Where, N_0_ = the number of nauplii taken; N_1_ = the number of nauplii alive.

### 2.9 Thrombolytic activity

Thrombolytic activity test performed using the method described by Prasad et al. [29]. As a stock solution, lyophilized streptokinase vial (1500000 IU) mixed adequately with 5 mL sterile distilled water from which appropriate dilution made. Venous blood was withdrawn (5 mL) from healthy volunteers (n = 6) without the history of anticoagulant therapy or an oral contraceptive. Then distributed (0.5 mL/tube) to each six previously weight microcentrifuge tubes (sterilized) and incubated to form the clot at 37 °C for 45 min. After the formation of the clot, completely removed the serum without disturbing the clot and each tube reweighed for calculating the clot weight. 100 μL extract (10 mg/mL) added to each tube having the pre-weighed clot. 100 μL streptokinase and 100 μL distilled water were added separately to the positive and negative control group. Incubation was done for 90 min at 37 °C and observed clot lysis. The released fluid was removed and reweighed the tube to calculate the difference in weight after clot disruption.

% of clot lysis = (weight of clot after remove of fluid/clot weight) × 100

### 2.10 Analgesic activity assay

#### 2.10.1 Acetic acid induced writhing inhibition test

The analgesic activity evaluated by the acetic acid-induced writhing test [30,31]. Before starting the test, all experimental animals were unfed for 2 h. The mice separated into ten groups (n = 5). As negative control, 1% Tween-80 solution at 10 mL/kg b.w. given orally and as a positive control (Diclofenac sodium) has been given at 25 mg/kg b.w., IP. Plant extracts (MEALT, NFALT, MEATS, and NFATS) administrate with a dose of 200 and 400 mg/kg b.w. by orally using gavage, respectively. Thirty minutes after administration, 0.7% acetic acid was injected into the mice intraperitoneally and record and count the number of writhing for 20 min.

#### 2.10.2 Formalin induced paw licking test

The analgesic activity was evaluated by the formalin-induced licking test [32] with the treatment of animals of each group (n = 5), as described in the acetic acid-induced writhing test. After 30 min of the administration, 20 μL formalin (2.5% v/V) was injected into the right hind paw just under the skin of the dorsal surface by a micro-syringe having a 26-gauge needle. The licking time of the first 5 min as early phase and then 15–30 min as late phase recorded.

### 2.11 Anti-diarrheal activities

#### 2.11.1 Castor oil-induced diarrhea

Antidiarrheal activities carried by the method Nwodo and Alumanah (1991) [33]. All experimental animals unfed for 24 h before starting the test. The mice separated into ten groups (n = 5). As negative control, 1% Tween-80 solution at 10 mL/kg b.w. given orally and as a positive control (loperamide) has been given at 5 mg/kg b.w., IP. Plant extract (MEATL, NFATL, MEATS, and NFATS) has been received orally by gavage in a dose of 200 and 400 mg/kg b.w., respectively. One hour after administration, 0.5 mL castor oil has been given orally and kept them in separate cages consist of adsorbent paper beneath. The feces were counted and observed every hour till 4 h for each mouse and replaced in every 1 h. The equation calculated the level of % inhibition of defecation:

$$\text{\% inhibition of defecation}=\;\frac{A-B}{A}\;\times 100$$

Where, A = average eradication feces number of the control group; B = average eradication feces number of the text group.

#### 2.11.2 Castor oil-induced gastrointestinal motility

This gastrointestinal motility experiment was carried out by the method described by Mascolo et al. [34] with the treatment of animals of each group (n = 5) as described in the castor oil-induced diarrhea. One hour after administration of treatment group, animals treated with 1 mL charcoal meal (10% charcoal in 5% gum acacia) administered orally to each mouse. One hour later of charcoal administration, the animals were sacrificed. The distance travel charcoal meal from the pylorus to caecum was determined and presented as the total length of the intestine in percentage. The following formulae used to express the percentage of inhibition and Peristalsis index

$$\%\;Inhibition=\;\frac{Distance\;travel\;by\;the\;control\left(cm\right)-Distance\;travel\;by\;the\;test\;groups\left(cm\right)\;}{Distance\;travel\;by\;the\;control\left(cm\right)}\;\;\;\times 100$$

### 2.12 Statistical analysis

Values were represented in Mean ± SEM (n = 5). ^a^ P < 0.05, ^b^ P < 0.01, ^c^ P < 0.001 and ^d^ P < 0.0001 indicated statistically significant in comparison to control group followed by unpaired *t*-test of one-way ANOVA (GraphPad Prism ver 7.0).

## 3 Results

### 3.1 Qualitative phytochemical screening

The qualitative phytochemical analysis of methanolic and n-hexane extract of *A. trilobata* leaves and stem showed the presence of alkaloid, carbohydrate, glycosides, flavonoids, phenols, flavonol, and saponins in all four extracts. In contrast, terpenoids only present in the methanolic extract of *A. trilobata* leaves. The phytochemical analysis in a qualitative manner summarized in Table 1.

**Table 1:** Comparative phytochemical screening of *A. trilobata* leaves and stem.

Qualitative Phytochemical analysis of *A. trilobata*				
Test Name	MEATL	NFATL	MEATS	NFATS
Alkaloid Mayer test	++	+	++	+
Carbohydrate Molisch's test	++	+	++	+
Glycosides	++	+	++	+
Flavonoids	++	++	+	++
Phenols	++	+	+	+
Flavonol	++	+	++	+
Terpenoids	+	–	–	–
Tannins	–	–	–	–
Saponins	+	–	+	+
Sterols	–	–	–	–
Quinones	–	–	–	–
Cholesterol	–	–	–	–
Proteins	–	–	–	–
Steroids	–	–	–	–
Starch	–	–	–	–

Here, ‘+ +’ or ‘+’: present; ‘-‘: absent.

MEATL: Methanolic extract of *A. trilobata* leaves*,* NFATL: n-hexane fraction of *A. trilobata* leaves, MEATS: Methanolic extract of *A. trilobata* stem and NFATS: n-hexane fraction of *A. trilobata* stem.

### 3.2 Antioxidant activity

#### 3.2.1 DPPH free radical scavenging activity

**Fig. 1:**
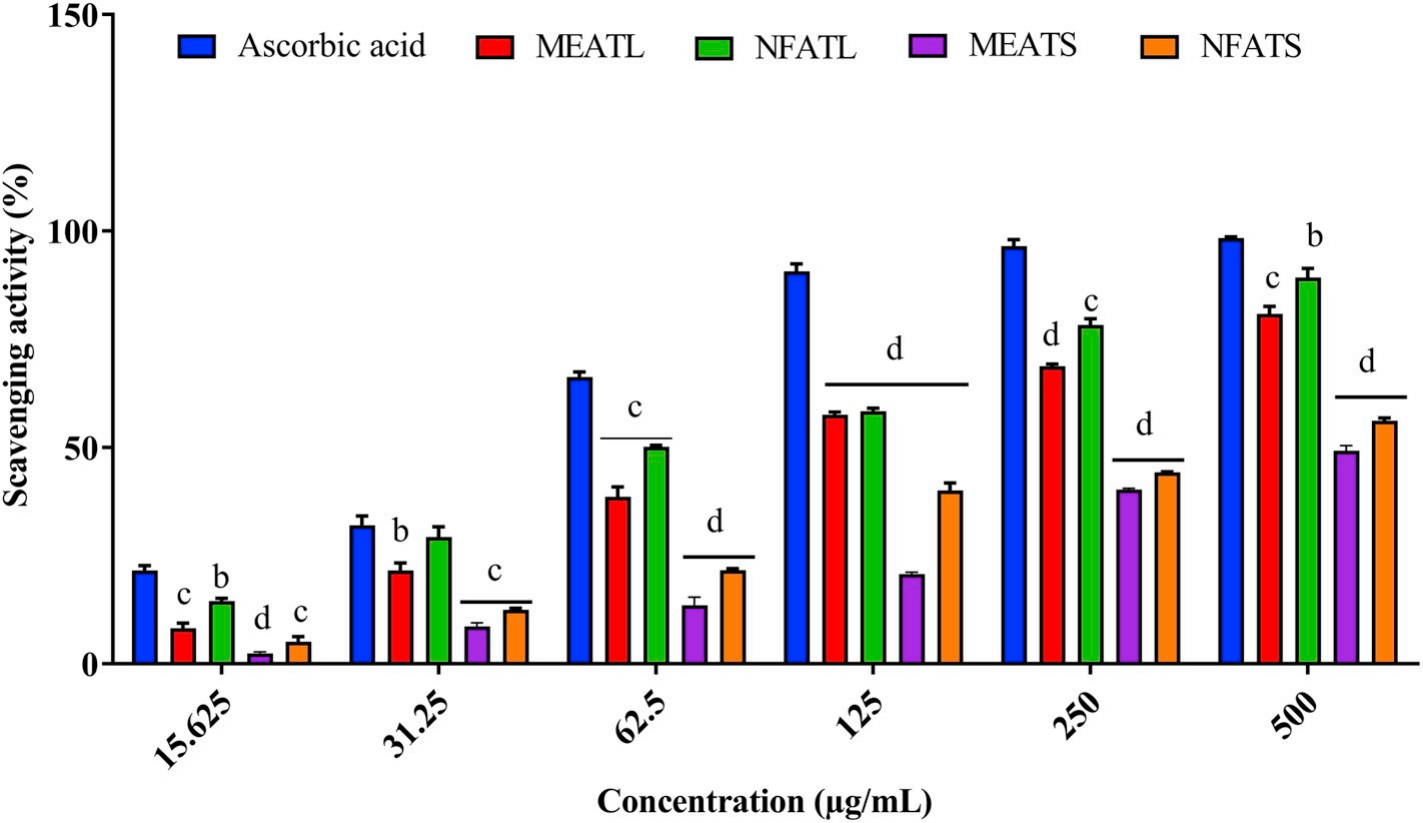
Percentage of radical scavenging activities the DPPH assay of *A. trilobata* fractions and standard drug at different concentrations. Values are represented in Mean ± SEM (n = 3). ^b^ P < 0.01, ^c^ P < 0.001 and ^d^ P < 0.0001 are statistically significant in comparison to ascorbic acid followed by unpaired *t*-test of one-way ANOVA (GraphPad Prism 7). MEATL: Methanolic extract of *A. trilobata* leaves*,* NFATL: n-hexane fraction of *A. trilobata* leaves, MEATS: Methanolic extract of *A. trilobata* stem and NFATS: n-hexane fraction of *A. trilobata* stem.

Table 2 and Fig. 1 were summarized the scavenging activity of DPPH assay and were in the following order: ascorbic acid > NFATL > MEATL > NFATS > MEATS. The antioxidant DPPH scavenging activity of *A. trilobata* fractions exhibited a significant (P < 0.05) manner inhibition in compared to standard drug ascorbic acid. Among all four extracts, NFATL showed the highest scavenging activity at (89.19%) at 500 μg/mL while at the same concentration ascorbic acid exhibited 98.33% scavenging activity. IC_50_ values calculated by the linear regression equation, whereas the IC_50_ values of ascorbic acid and NFATL were 36.32 and 139.65 μg/mL, respectively.

**Table 2:** IC_50_ values with regression equation for *A. trilobata* fractions with reference to ascorbic acid.

IC_50_ values (μg/mL) of radical scavenging		
Chemicals/Plant extracts	IC_50_	Regression equation
Ascorbic acid	36.32	y = 0.1374x + 45.009; R^2^ = 0.5688
MEATL	194.77	y = 0.133x + 24.095; R^2^ = 0.7687
NFATL	139.65	y = 0.1355x + 31.078; R^2^ = 0.7827
MEATS	454.09	y = 0.095x + 6.8611; R^2^ = 0.9064
NFATS	372.95	y = 0.096x + 14.197; R^2^ = 0.798

MEATL: Methanolic extract of *A. trilobata* leaves*,* NFATL: n-hexane fraction of *A. trilobata* leaves, MEATS: Methanolic extract of *A. trilobata* stem and NFATS: n-hexane fraction of *A. trilobata* stem.

#### 3.2.2 Reducing power activity

**Fig. 2:**
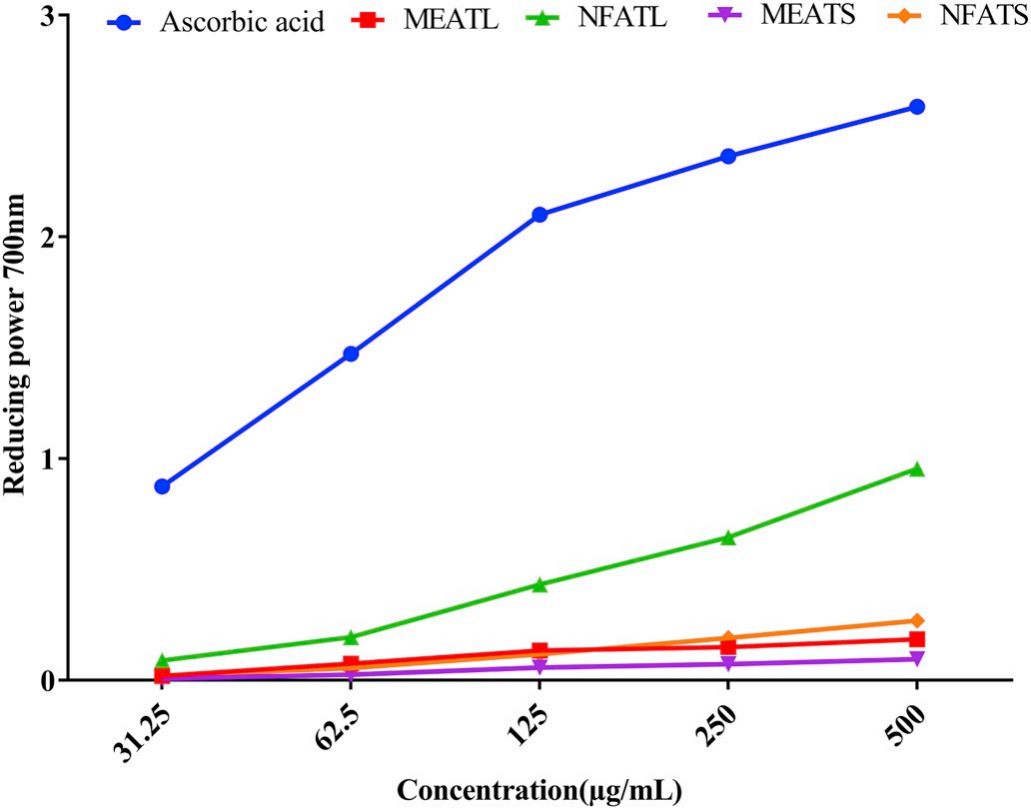
Reducing power of *A. trilobata* fractions and standard drug at different concentrations. MEATL: Methanolic extract of *A. trilobata* leaves*,* NFATL: n-hexane fraction of *A. trilobata* leaves, MEATS: Methanolic extract of *A. trilobata* stem and NFATS: n-hexane fraction of *A. trilobata* stem.

In Fig. 2, the dose-response curve for reducing power activity for *A. trilobata* fractions summarized (31.25–500 μg/mL). Reducing power will be increased with the increase of the concentration of the samples. The orders for reducing power activity were as followed: ascorbic acid > NFATL > NFATS > MEATL > MEATS. NFATL exhibited higher reducing power activity 0.955 at 500 μg/mL.

#### 3.2.3 Total phenolic, flavonoid and flavonol contents

Quantitative analysis of antioxidant relevant phytochemicals total phenol content, total flavonoid content, and total flavonol content of *A. trilobata* (AT) fractions (500 μg/mL) summarized in Table 3 along with their regression equation. The orders for antioxidant relevant phytochemicals activity were as followed: NFATL > MEATL > NFATS > MEATS. NFATL showed the highest total phenol content (69.68 ± 0.67 mg GAE/g AT), total flavonoid content (53.69 ± 0.35 mg QE/g AT), and total flavonol content (153.26 ± 0.75 mg GAE/g AT) followed by MEATL, NFATS, and MEATS.

**Table 3:** Quantitative analysis of antioxidant relevant phytochemicals total phenol content, total flavonoid content and total flavonol content of *A. trilobata* (AT) fractions (500 μg/mL).

Plant extracts	Total phenol content (mg GAE/g AT)	Total flavonoid content (mg QE/g AT)	Total flavonol content (mg GAE/g AT)
MEATL	40.19 ± 0.69	46.67 ± 0.23	118.91 ± 0.99
NFATL	69.68 ± 0.67	53.69 ± 0.35	153.26 ± 0.75
MEATS	13.69 ± 1.85	10.62 ± 0.55	26.00 ± 1.94
NFATS	23.44 ± 1.04	12.85 ± 0.28	37.19 ± 1.19
Regression equation	y = 0.0039x + 0.0406 R^2^ = 0.9981	y = 0.0102x - 0.0637 R^2^ = 0.9693	y = 0.0039x + 0.0406 R^2^ = 0.9981

MEATL: Methanolic extract of *A. trilobata* leaves*,* NFATL: n-hexane fraction of *A. trilobata* leaves, MEATS: Methanolic extract of *A. trilobata* stem and NFATs: n-hexane fraction of *A. trilobata* stem.

Each value in the table is represented as mean ± SEM (n = 3).

### 3.3 Brine shrimp cytotoxicity

**Fig. 3:**
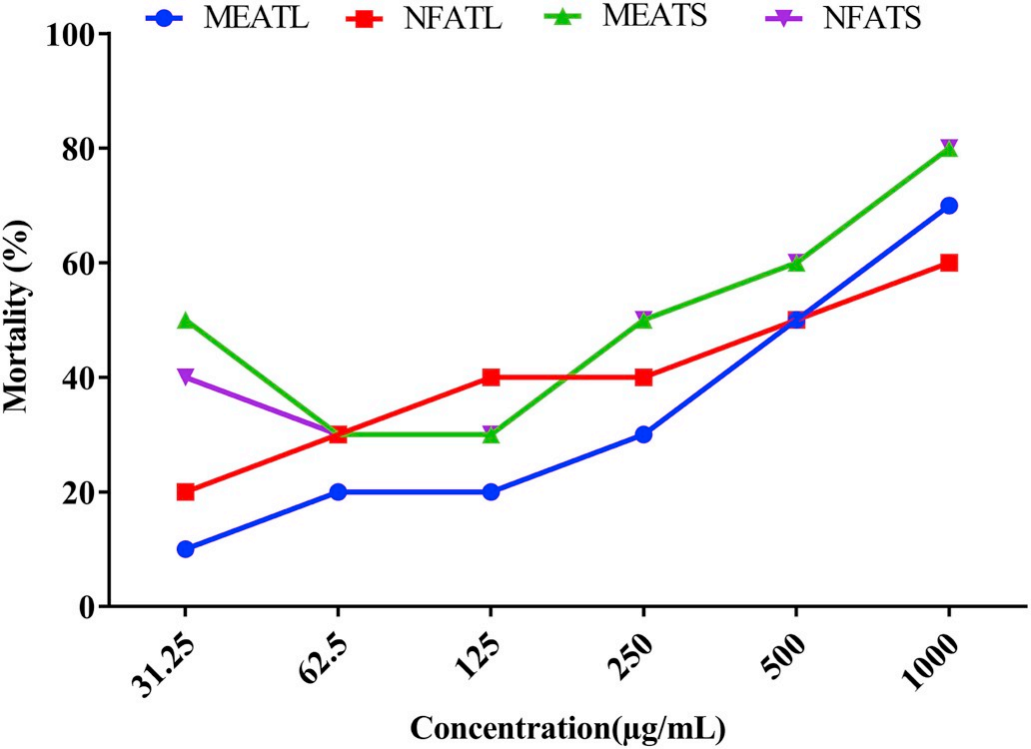
Percentage of mortality of brine shrimp of *A. trilobata* fractions and standard drug at different concentrations. MEATL: Methanolic extract of *A. trilobata* leaves*,* NFATL: n-hexane fraction of *A. trilobata* leaves, MEATS: Methanolic extract of *A. trilobata* stem and NFATs: n-hexane fraction of *A. trilobata* stem.

Table 4 and Fig. 3 summarized the LC_50_ values with the regression equation and the percentage of mortality of *A. trilobata* fractions, where no extract found to be toxic in comparison to positive control vincristine sulfate (2.16 μg/mL). The *A. trilobata* fractions exhibited moderately (MEATS = 328 μg/mL) to weakly toxic (NFATL = 616.85 μg/mL) LC_50_ observed.

**Table 4:** LC_50_ values with regression equation for *A. trilobata* fractions with reference to vincristine sulfate.

LC_50_ values (μg/mL) of Brine shrimp		
Chemicals/Plant extracts	LC_50_	Regression equation
Vincristine sulfate	2.16	y = 7.6315x + 33.536, R^2^ = 0.8585
MEATL	607.70	y = 0.0596x + 13.781; R^2^ = 0.9629
NFATL	616.85	y = 0.0346x + 28.657; R^2^ = 0.821
MEATS	328.02	y = 0.0455x + 35.075; R^2^ = 0.7897
NFATS	362.00	y = 0.0498x + 31.972; R^2^ = 0.9053

MEATL: Methanolic extract of *A. trilobata* leaves*,* NFATL: n-hexane fraction of *A. trilobata* leaves, MEATS: Methanolic extract of *A. trilobata* stem and NFATs: n-hexane fraction of *A. trilobata* stem.

### 3.4 Thrombolytic activity

**Fig. 4:**
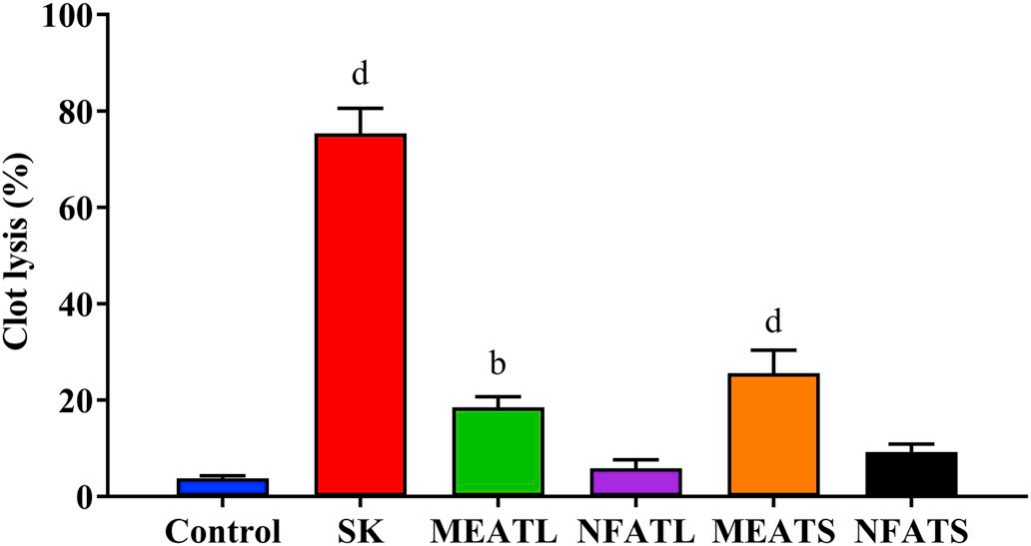
Percentage of clot lysis of human blood by *A. trilobata* fractions and standard drug. Values are represented in Mean ± SEM (n = 6). ^b^ P < 0.01 and ^d^ P < 0.0001 are statistically significant in comparison to negative control (water) followed by unpaired *t*-test of one-way ANOVA (GraphPad Prism 7). SK: streptokinase, MEATL: Methanolic extract of *A. trilobata* leaves*,* NFATL: n-hexane fraction of *A. trilobata* leaves, MEATS: Methanolic extract of *A. trilobata* stem and NFATS: n-hexane fraction of *A. trilobata* stem.

The thrombolytic activity of methanolic and n-hexane extract of *A. trilobata* leaves and stem summarized in Fig. 4. The MEATS showed the highest percentage of clot lysis (25.58 ± 4.76%, P < 0.0001) in comparison to negative control water (3.78 ± 0.49%), whereas the standard drug streptokinase exhibited 75.35 ± 5.21% (P < 0.0001). The orders for percentage of clot lysis were as followed: streptokinase > MEATS > MEATL > NFATS > NFATL > water.

### 3.5 Analgesic activity

#### 3.5.1 Acetic acid induced writhing inhibition test

The methanolic and n-hexane extract of *A. trilobata* leaves stem at a dose of 200 and 400 mg/kg exhibited a significant decreased in the number of writhing. After induced of acetic acid, the NFATL (200 and 400 mg/kg) showed 20 and 17 writhing in per 20 min while the MEATL writhing (200 and 400 mg/kg) counted 23.33 and 14.33 in per 20 min; whereas the standard drug diclofenac Na (10 mg/kg) showed 12.33 writhing/20 min. The highest percentage of inhibition (57.85%) observed at MEATL (400 mg/kg), whereas Diclofenac Na showed 63.74% (Table 5).

**Table 5:** Effect of *A. trilobata* fractions on acetic acid induced writhing response on Swiss albino mice.

Acetic acid induced writhing inhibition test		
Treatments (mg/kg)	Number of writhing	Inhibition (%)
Control	34.00 ± 3.00	–
Diclofenac Na 10	12.33 ± 0.88 ^d^	63.74
MEATL 200	23.33 ± 1.76 ^b^	31.38
MEATL 400	14.33 ± 0.88 ^c^	57.85
NFATL 200	20.00 ± 2.00 ^b^	41.18
NFATL 400	17.00 ± 1.00 ^c^	50.00
MEATS 200	26.67 ± 1.45 ^a^	21.56
MEATS 400	21.33 ± 0.88 ^b^	37.26
NFATS 200	26.33 ± 1.45 ^a^	22.56
NFATS 400	23.67 ± 1.45 ^b^	30.38

Values are represented in Mean ± SEM (n = 5). ^a^ P < 0.05, ^b^ P < 0.01, ^c^ P < 0.001 and ^d^ P < 0.0001 are statistically significant in comparison to Diclofenac Na followed by unpaired *t*-test of one-way ANOVA (GraphPad Prism 7).

MEATL: Methanolic extract of *A. trilobata* leaves*,* NFATL: n-hexane fraction of *A. trilobata* leaves, MEATS: Methanolic extract of *A. trilobata* stem and NFATS: n-hexane fraction of *A. trilobata* stem.

#### 3.5.2 Formalin induced analgesic test

The effect of formalin-induced licking tests for analgesic activity summarized in Table 6. The extract of methanolic and n-hexane extract of *A. trilobata* leaves and stem at a dose of 200 and 400 mg/kg exhibited a significant depleted manner decreased in both early and late phases. But, MEATL (400 mg/kg) showed significant (P < 0.0001) percentage of inhibition in both early and late phases (60.83% and 61.65%, respectively), which were almost similar to standard drug diclofenac Na (68.68 and 63.16%).

**Table 6:** The effect of *A. trilobata* fractions in Swiss albino mice to evaluate the analgesic activity by formalin induced licking response.

Formalin induced licking test				
Treatment (mg/kg)	Early phase (0–5 min)	Inhibition (%)	Late phase (15–30 min)	Inhibition (%)
Control	55.33 ± 4.33	–	44.33 ± 0.33	–
Diclofenac Na10	17.33 ± 0.33 ^d^	68.68	16.33 ± 0.33 ^d^	63.16
MEATL 200	36.67 ± 3.18 ^b^	33.72	29.33 ± 2.03 ^d^	33.84
MEATL 400	21.67 ± 1.76 ^d^	60.83	17.00 ± 1.52 ^d^	61.65
NFATL 200	43.00 ± 1.73 ^a^	22.28	32.33 ± 0.33 ^d^	27.07
NFATL 400	27.33 ± 2.03 ^c^	50.61	19.67 ± 1.76 ^d^	55.63
MEATS 200	31.67 ± 1.76 ^c^	42.76	22.00 ± 2.08 ^d^	50.37
MEATS 400	27.00 ± 2.65 ^c^	51.20	25.66 ± 1.20 ^d^	42.0
NFATS 200	34.33 ± 2.73 ^b^	37.95	24.67 ± 1.20 ^d^	44.35
NFATS 400	28.67 ± 1.20 ^c^	48.18	26.33 ± 1.45 ^d^	40.60

Values are represented in Mean ± SEM (n = 5). ^a^ P < 0.05, ^b^ P < 0.01, ^c^ P < 0.001 and ^d^ P < 0.0001 are statistically significant in comparison to Diclofenac Na followed by unpaired *t*-test of one-way ANOVA (GraphPad Prism 7). MEATL: Methanolic extract of *A. trilobata* leaves*,* NFATL: n-hexane fraction of *A. trilobata* leaves, MEATS: Methanolic extract of *A. trilobata* stem and NFATS: n-hexane fraction of *A. trilobata* stem.

### 3.6 Anti-diarrheal activity

#### 3.6.1 Castor oil-induced diarrhea in mice

The effect of castor oil-induced diarrhea in mice by *A. trilobata* fractions summarized in Table 7. In comparison to the negative control, the extract showed significant inhibition in both diarrhea and defecation phase in a dose-dependent manner while the MEATL 200, 400 mg/kg showed extremely significant inhibition in defecation (P < 0.0001) and diarrhea (P < 0.001) which is higher than the standard drug loperamide.

**Table 7:** The effect of *A. trilobata* fractions on castor oil induced diarrhea in mice (feces count).

Castor oil induced diarrhea test				
Treatment	Total number of feces	% of Inhibition of defecation	Total number of diarrheal feces	% Inhibition of diarrhea
Control	14.60 ± 0.87	–	6.40 ± 0.81	–
Loperamide 5	5.40 ± 0.24 ^d^	63.01	2.20 ± 0.20 ^c^	65.63
MEATL 200	4.33 ± 0.33 ^d^	70.34	1.75 ± 0.14 ^c^	72.66
MEATL 400	2.45 ± 0.10 ^d^	83.22	1.57 ± 0.32 ^c^	75.47
NFATL 200	6.83 ± 0.36 ^d^	53.21	1.9 ± 0.15 ^c^	70.31
NFATL 400	3.50 ± 0.52 ^d^	76.02	1.42 ± 0.08 ^c^	77.81
MEATS 200	7.08 ± 1.08 ^c^	51.51	3.17 ± 0.3 ^b^	50.47
MEATS 400	7.65 ± 0.78 ^c^	47.60	1.92 ± 0.44 ^c^	70.00
NFATS 200	9.33 ± 0.88 ^c^	36.09	2.58 ± 0.22 ^c^	59.69
NFATS 400	7.58 ± 0.3 ^c^	48.08	2.17 ± 0.17 ^c^	66.09

Values are represented in Mean ± SEM (n = 5). ^b^ P < 0.01, ^c^ P < 0.001 and ^d^ P < 0.0001 are statistically significant in comparison to Tween-80 (Control) followed by unpaired *t*-test of one-way ANOVA (GraphPad Prism 7).

MEATL: Methanolic extract of *A. trilobata* leaves*,* NFATL: n-hexane fraction of *A. trilobata* leaves, MEATS: Methanolic extract of *A. trilobata* stem and NFATS: n-hexane fraction of *A. trilobata* stem.

#### 3.6.2 Castor oil induced intestinal motility

The effect of castor oil-induced intestinal motility by *A. trilobata* fractions summarized in Table 8. In comparison to the negative control, the extract showed a significant decrease in peristalsis index while the NFATL 400 mg/kg showed significant reduced (48.79 ± 1.94; P < 0.0001), whereas the standard drug loperamide showed 43.04 ± 2.79 (P < 0.0001) percentage of decrease. Of those, 400 mg/kg dose of NFATL exhibited the highest percentage inhibition (41.22%) of intestinal motility, whereas the standard drug loperamide (48.09%).

**Table 8:** The effect of *A. trilobata* fractions with reference to Loperamide on intestinal motility in mice by using charcoal as a marker.

Castor oil-induced intestinal motility test				
Treatment	Total Length of Intestine (cm)	Distance Travel by Charcoal (cm)	Peristalsis Index (%)	Inhibition (%)
Control	50.33 ± 0.33	43.66 ± 2.91	86.69 ± 5.23	–
Loperamide 5	52.66 ± 0.33 ^c^	22.66 ± 1.45 ^c^	43.04 ± 2.79 ^d^	48.09
MEATL 200	53.67 ± 0.88 ^b^	36.00 ± 1.53 ^a^	67.11 ± 3.00 ^b^	17.56
MEATL 400	57.33 ± 1.20 ^c^	29.66 ± 1.76 ^b^	51.71 ± 2.59 ^c^	32.06
NFATL 200	54.33 ± 1.45 ^a^	35.00 ± 1.53 ^a^	67.37 ± 1.51 ^b^	19.85
NFATL 400	52.66 ± 1.45	25.66 ± 0.88 ^c^	48.79 ± 1.94 ^d^	41.22
MEATS 200	48.66 ± 0.67 ^a^	30.00 ± 1.53 ^b^	61.58 ± 2.29 ^b^	31.29
MEATS 400	49.00 ± 0.58	29.67 ± 1.15 ^b^	60.60 ± 5.02 ^b^	32.06
NFATS 200	53.00 ± 1.15 ^a^	31.00 ± 1.15 ^b^	58.50 ± 1.91 ^c^	29.01
NFATS 400	52.33 ± 3.53	28.33 ± 1.76 ^b^	54.17 ± 3.53 ^c^	35.11

Values are represented in Mean ± SEM (n = 5). ^a^ P < 0.05, ^b^ P < 0.01, ^c^ P < 0.001 and ^d^ P < 0.0001 are statistically significant in comparison to Tween-80 (control) followed by unpaired *t*-test of one-way ANOVA (GraphPad Prism 7).

MEATL: Methanolic extract of *A. trilobata* leaves*,* NFATL: n-hexane fraction of *A. trilobata* leaves, MEATS: Methanolic extract of *A. trilobata* stem and NFATS: n-hexane fraction of *A. trilobata* stem.

## 4 Discussion

Phytochemical analysis of plant extract revealed the physiological activities as well as therapeutic activities also [35]. The phytochemical analysis of *A. trilobata* shows the presence of several secondary metabolites, whereas alkaloids are a particular group of secondary nitrogenous compounds which used to treat several human and animal disorder during middle age [36]. Another major phytochemical group is flavonoids, which used to treat cardiovascular diseases, cancer, and anti-inflammatory. Flavonoids usually take in dietary [37]. Presence of phenol in plants, having a contribution in physiological or biological elements of the plant [38].

Plants are an excellent source for natural antioxidants, whereas several phytochemicals have antioxidant properties. Their primary action is to ensure the protection against oxidative stress from free radicals [39,40]. Free radicals involve in several diseases like coronary infarction, atherosclerosis, neurodegenerative diseases and cancer [41]. Due to the potent antioxidant activity of polyphenolic substances (flavonoids and phenolic acids) in a biological system, are of interest [42,43]. According to literature, oxidative stress inhibited by quercetin [44] and gallic acid regulates the reactive species generation and improved a higher ratio of glutathione/oxidized glutathione [45]. Generally, the antioxidant activity was enhanced synergistically by antiradical plant phenolic substances [46]. Study reports, using of these phytochemicals substances as chemical agents (e.g., anti-inflammatory, antioxidant, anticancer), which may be useful to prevent the reactive oxygen species and antioxidant defense system [47]. In our study, all of this antioxidant relevant phytochemicals (total phenol content, total flavonoid content, and total flavonol content) of *A. trilobata* exhibited higher antioxidant activity in this order: NFATL > MEATL > NFATS > MEATS which also correlated with IC_50_ values of DPPH scavenger. At the same time, the extract also exhibited a significant decrease in free radical scavenging assay with the correlation of concentration. The brine shrimp lethality bioassay is a safe, effective, and economical method to determine the bioactivity of plant products [48]. The correlation between brine shrimp lethality bioassay and *in vitro* depletion of solid tumors in a human was introduced by the National Cancer Institute (NCI, USA). It is useful because it used as a pre-screening test for antitumor research [49]. LC_50_ and toxicity are inversely proportional, whereas the higher the toxicity, the lower will be the LC_50_. The value of LC_50_ over 1000 μg/mL considered to be non-toxic, ranging from 500 to 1000 μg/mL is weakly toxic, 100–500 μg/mL is moderately toxic while less than 100 μg/mL is considered as highly toxic [50]. In our study, no *A. trilobata* extracts found to be toxic in comparison to Vincristine sulfate, while a moderately (MEATS = 328 μg/mL) to weakly toxic (NFATL = 616.85 μg/mL) LC_50_ observed. The observed cytotoxic activity may be present of alkaloid phytochemical in the extract [51], which required further study to evaluate the cytotoxic compounds.

The available drugs in the market for thrombolytic, especially streptokinase, convert plasminogen to plasmin and increased lysis of the clot. The researcher revealed that flavonoids (plant metabolites) affect embolus and cardiovascular disease by interfering with platelet activation which is a risk factor for a cardiac disorder [52,53]. Our study suggests that the MEATS and MEATL could be a potential source to inhibit the clot formation. The current finding of *A. trilobata* through clot lysis in blood supports the earlier study on different plant species of the Passifloraceae family [54].

Nociception triggered or activated by mediators, which cause inflammation, edema, and diapedesis or leukocyte eruption [55]. Acetic acid-induced in mice is a behavioral analgesia mediated observation where IP administrations of acetic acid produce increase prostaglandin in the fluid of peritoneal [56]. By inhibiting the prostaglandin synthesis by any chemical substances which lower the number of writhing mentioned as analgesia. In contrast, central and peripheral antinociceptive pain is a biphasic method for formalin-induced licking test. The early phase and late phase is neurogenic and inflammatory pain response, respectively. Narcotics inhibit both phases while the non-steroidal anti-inflammatory drug (NSAID) inhibits the late phase [57]. In an acetic acid test, the extract of *A. trilobata* showed a significant dose-dependent manner percentage of inhibition in the nociceptive nerve, whereas the methanolic and n-hexane fraction of *A. trilobata* leaves showed the highest percentage of inhibition. Similarly, the administration of methanolic and n-hexane fraction of *A. trilobata* leaves and stem showed significant inhibition in both phases in a depleted manner, whereas the MEATL (400 mg/kg) showed the highest percentage of inhibition. The result showed significant inhibition in analgesic activity might be for the presence of alkaloids [58]. At the same time, the saponins and flavonoids phytochemicals are responsible for the anti-inflammatory properties of medicinal plants [59,60].The analgesic activity might also show because of the traditional used in pain sensation [15,16].

In antidiarrheal screening, castor oil cause water and electrolyte penetrability changes in the intestinal mucosal layers, bringing about liquid and watery luminal content that rapidly eliminate by intestines [61]. Ricinoleic acid is a natural laxative which is the active metabolite of castor oil, used in evaluating castor oil-induced diarrheal activity. Castor oil act on the small intestine to change the action of smooth muscle GI [62]. In our study, both models for antidiarrheal showed extremely significant inhibition of diarrhea. In contrast, the castor oil-induced diarrhea model exhibited extremely significant inhibition in defecation (P < 0.0001) and diarrhea (P < 0.001) by MEATL (200, 400 mg/kg), which is higher than the standard drug loperamide. Though the extremely significant activity in the castor oil-induced diarrhea model, it did not possess similar activity in castor oil-induced intestinal motility test. In contrast, the NFATL showed the maximum inhibition of intestinal motility. But, the finding of both models suggesting that the *A. trilobata* could be a potential source for diarrheal treatment, which proven the traditional used in stomach trouble [15,16].

## 5 Conclusion

The study reported that *A. trilobata* could be a potential source for antioxidant, cytotoxic, thrombolytic, analgesic, antidiarrheal activity due to the presence of secondary metabolites (e.g., alkaloid, flavonoid, phenol). Furthermore, studies highly recommended in identifying the mechanism of *A. trilobata* because there are no scientific reports related to biological activity.

## Ethical approval

The study approved by the Institutional Animal Ethical Committee, Department of Pharmacy, International Islamic University Chittagong, Bangladesh according to governmental guidelines under the reference of Pharm/PND/150/20–2019.

## 6 Consent for publication

All authors have agreed to publish all materials belongs to this article.

## 7 Availability of data and materials

The datasets used and/or analyzed during the current study are available from the corresponding author on reasonable request.

## Funding

This work is conducted with the individual funding of all authors.

## CRediT authorship contribution statement

**Niloy Barua:** Conceptualization, Data curation, Formal analysis, Investigation, Methodology, Writing - original draft. **Md Arfin Ibn Aziz:** Conceptualization, Data curation, Formal analysis, Investigation, Methodology, Writing - original draft. **Abu Montakim Tareq:** Conceptualization, Data curation, Formal analysis, Investigation, Methodology, Writing - original draft. **Mohammed Aktar Sayeed:** Funding acquisition, Project administration, Resources, Supervision. **Najmul Alam:** Investigation, Methodology, Validation, Visualization. **Nobi ul Alam:** Investigation, Methodology, Validation, Visualization. **Mohammad Amran Uddin:** Investigation, Methodology, Validation, Visualization. **Chadni Lyzu:** Resources, Software, Validation, Visualization, Writing - original draft. **Talha Bin Emran:** Funding acquisition, Project administration, Resources, Supervision, Writing - review & editing.

## Declaration of competing interest

The authors declare that they have no known competing financial interests or personal relationships that could have appeared to influence the work reported in this paper.
